# Molecular dynamics re-refinement of two different small RNA loop structures using the original NMR data suggest a common structure

**DOI:** 10.1007/s10858-012-9642-5

**Published:** 2012-06-20

**Authors:** Niel M. Henriksen, Darrell R. Davis, Thomas E. Cheatham III

**Affiliations:** Department of Medicinal Chemistry, College of Pharmacy, University of Utah, 2000 East 30 South Skaggs 201, Salt Lake City, UT 84112 USA

**Keywords:** RNA structure, Molecular dynamics, Residual dipolar coupling restraints, Bulge structure, Force fields, Ion binding

## Abstract

Restrained molecular dynamics simulations are a robust, though perhaps underused, tool for the end-stage refinement of biomolecular structures. We demonstrate their utility—using modern simulation protocols, optimized force fields, and inclusion of explicit solvent and mobile counterions—by re-investigating the solution structures of two RNA hairpins that had previously been refined using conventional techniques. The structures, both domain 5 group II intron ribozymes from yeast ai5γ and *Pylaiella littoralis*, share a nearly identical primary sequence yet the published 3D structures appear quite different. Relatively long restrained MD simulations using the original NMR restraint data identified the presence of a small set of violated distance restraints in one structure and a possibly incorrect trapped bulge nucleotide conformation in the other structure. The removal of problematic distance restraints and the addition of a heating step yielded representative ensembles with very similar 3D structures and much lower pairwise RMSD values. Analysis of ion density during the restrained simulations helped to explain chemical shift perturbation data published previously. These results suggest that restrained MD simulations, with proper caution, can be used to “update” older structures or aid in the refinement of new structures that lack sufficient experimental data to produce a high quality result. Notable cautions include the need for sufficient sampling, awareness of potential force field bias (such as small angle deviations with the current AMBER force fields), and a proper balance between the various restraint weights.

**Electronic supplementary material:**

The online version of this article (doi:10.1007/s10858-012-9642-5) contains supplementary material, which is available to authorized users.

## Introduction

Molecular dynamics (MD) simulations are often used with restraints derived from crystallography or nuclear magnetic resonance (NMR) experiments for the end-stage atomistic refinement of biological macromolecular structures (Clore et al. 1985; Brunger et al. 1987a, b; Nilges 1996; Brunger and Adams 2002). Quite commonly, rather quick and standard MD refinement protocols are employed using codes such as X-PLOR (Brunger 1992), XPLOR-NIH (Schweiters et al. 2003, 2006) or CNS (Brunger et al. 1998; Brunger 2007). A refinement protocol might initiate a search for restraint-compatible structures via simulated annealing or distance geometry methods followed by very short (20–200 picosecond) gas-phase MD simulations with applied restraints to further relax and refine the structures. In these refinement protocols, often the force field—specifically the molecular mechanical parameters and force constants that define the covalent connectivity and atomic pair interactions—is rather simplified or crude and the MD simulations are performed in vacuo in the absence of solvent and mobile counter-ions. Despite the limitations of these simplified force fields, excellent results are generally obtained given a sufficiently robust set of experimentally derived data. This latter point is somewhat obvious noting that, if sufficient data from experiment has been collected to define the structure, the force field should not strongly influence or bias the results as the structure should be largely determined by the experimental data. However, when the experimental data is sparse, the structure is dynamic, or when solvent and mobile ions may be critically important elements of the structure, the arguably simple force fields and/or absences of experimental restraint data may lead to “loose” structure ensembles. These loose ensembles may suggest larger ranges of motion where data was absent and/or populate anomalous structures leading to an incorrect interpretation of the structure. A logical step to make up for missing data is to apply optimized biomolecular force fields in MD simulations with explicit solvent and modern simulation protocols. A relevant example involves the refinement of nucleic acid structures from NMR data, particularly in the absence of residual dipolar coupling (RDC) information, where long-range restraint information is absent (Konerding et al. 1999). If the force field and simulation protocols are reasonably robust and ideally experimentally validated, they together with the experimentally derived restraint information should provide a better representation of the structure. Although NMR refinement using modern MD simulation protocols with experimentally derived restraints, optimized force fields for proteins and nucleic acids, and explicit solvent suggests this to be true (Prompers et al. 1995; Kordel et al. 1997; Linge and Nilges 1999; Gouda et al. 2001; Spronk et al. 2002; Xia et al. 2002; Linge et al. 2003), such methods are not widely or routinely applied. For example, in recent years only a handful of NMR structures have been refined in explicit solvent using optimized force fields and modern simulation protocols, and these include the structure elucidation of a peptide (Dolenc et al. 2010), an RNA hairpin (Nozinovic et al. 2010), a DNA naphthalimide adduct (Rettig et al. 2010), a designed metalloprotein (Calhoun et al. 2008), and an RNA receptor/ligand complex (Paulsen et al. 2010a).

Although refinement of NMR structures using modern force fields and simulation protocols, including explicit solvent, appears to produce structures that provide excellent fits to the experimental data, these approaches are not without limitation. Complications relate to dynamics, the time scales of the dynamics, and whether these motions are even accessible on the timescale sampled during the molecular dynamics refinement. Moreover, RNA may populate multiple conformations under the given set of experimental conditions (Al-Hashimi and Walter 2008; Hall 2008; Baird and Ferre-D’Amare 2010; Solomatin et al. 2010; Stelzer et al. 2011). With traditional refinement, high restraint weights may lead to structural representations that are too tight, that hide dynamics, and that limit potential transformations between multiple conformations (James 2001). Essentially, an average structure will be found that may not entirely satisfy all of the experimental data. Procedures such as time-averaged restraints (Torda et al. 1990; Pearlman and Kollman 1991; Schmitz et al. 1993), selective enforcement of restraints over time (Gorler et al. 2000), or ensemble based refinement methods (Bonvin and Brunger 1995; Schwieters and Clore 2007) may help mitigate these issues. However, these methods will further depend on the reliability of the force field representation to correctly sample the accessible conformational space. Ultimately, given a reliable and validated force field, the molecular dynamics simulations without experimental restraints starting from the refined NMR structure should provide an accurate representation of the structure and dynamics over the simulation time scale. However, the force fields are not yet fully reliable, especially in the treatment of RNA (McDowell et al. 2007; Besseova et al. 2009; Hashem and Auffinger 2009; Banas et al. 2010; Deng and Cieplak 2010). Therefore, at present, there needs to be a careful balance between the relative weights of the force field compared to the experimental or structural restraints, with further care levied to understand the limitations of the force fields and implications of specific restraint choices.

In this work we further assess the reliability of more detailed MD structure refinement protocols through the re-refinement of two similar RNA molecules. As part of our larger force field assessment efforts, we have been investigating a variety of RNA structures in free, unrestrained MD simulation to better understand the reliability and flaws of the AMBER nucleic acid force fields (Cornell et al. 1995; Cheatham et al. 1999; Wang et al. 2000; Perez et al. 2007b; Banas et al. 2010; Yildirim et al. 2010; Zgarbova et al. 2011) as compared to available experimental data. Ideal RNA structures for our investigation include those which display some non-canonical structure (*i.e.*, non-helical structure since the AMBER force fields appear to do a reasonable job of modeling nucleic acid helices (Csaszar et al. 2001; Reblova et al. 2003; Beveridge et al. 2004; Dixit et al. 2005; Perez et al. 2007a; Ditzler et al. 2010; Lavery et al. 2010)) and importantly, structures where detailed NMR restraint information is available including NOE derived distance, J-coupling and RDC restraints. Our explorations led us to two published RNA structures that have nearly identical primary sequence, yet the published 3D structures differ significantly.

**Fig. 1:**
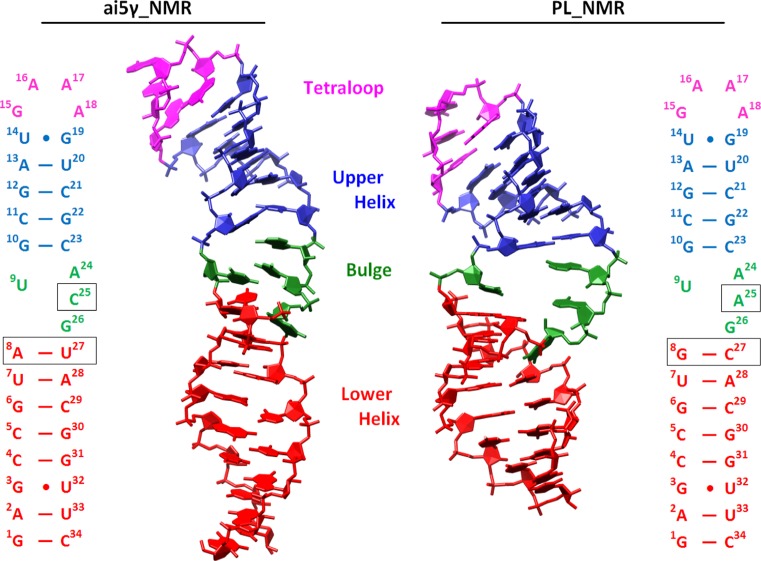
Comparison of the secondary and 3D structures for domain 5 of the Group II Intron from yeast ai5γ (ai5γ_NMR, PDB code 1R2P, *left*) and *Pylaiella littoralis* (PL_NMR, PDB code 2F88, *right*). The structural elements are indicated by color: tetraloop (*pink*), upper helix (*blue*), bulge (*green*), lower helix (*red*). Differences in sequence are boxed

A comprehensive MD investigation of these two previously refined RNA structures from the PDB (Kouranov et al. 2006) was performed, specifically on structures with the PDB codes of 1R2P (Sigel et al. 2004) and 2F88 (Seetharaman et al. 2006). These structures consist of a 34 residue segment derived from domain 5 (D5) of the group IIB intron ribozyme in yeast ai5γ (Sigel et al. 2004) (ai5γ-D5) and *Pylaiella littoralis* (Seetharaman et al. 2006) (PL-D5), respectively. Domain 5 serves an essential role in the core of the intron structure and contains the most important residues for catalysis (Keating et al. 2010). The primary sequence of ai5γ-D5 and PL-D5 is mostly identical, except for three residues (Fig. 1, noting the different boxed residues 8, 25, and 27). The structural elements of both D5’s include a lower helix joined by a bulge region to an upper helix that is capped by a GAAA tetraloop (shown in Fig. 1 in red, green, blue, and pink, respectively). Residues A2, G3, and C4 form what is known as the catalytic triad; this is a highly conserved region of interest noted for forming tertiary interactions and interactions with Mg^2+^. According to the published structures, the conformation of the bulge region differs depending both on the sequence and experimental method (NMR vs. X-ray crystallography), while the lower helix, upper helix, and tetraloop features are all very similar. The bulge conformation as reported in the earlier crystal structure of ai5γ-D5 (PDB: 1KXK) (Zhang and Doudna 2002) shows G26 forming a wobble pair with U9, while A24 and C25 are unpaired and opened away from the helix. In contrast, the NMR structures of ai5γ-D5 and PL-D5 (referred to as ai5γ_NMR and PL_NMR from here on) both suggest that G26 is in a *syn* conformation that protrudes into the major groove. However the positioning of the other bulge residues differ between the two NMR structures: while most of the ai5γ_NMR ensemble structures show residues 24 and 25 stacked into a narrow helix above U9, the PL_NMR structures show residues 24 and 25 in one of two conformations opposite, but not directly interacting with, U9. Both of these conformations of PL_NMR show a very wide bulge region accommodating A25 either stacked into the helix below A24 (as is shown in Fig. 1, right) or packed against the minor groove out of the helix. The differences in these bulge structures are highlighted in the supporting information using molecular graphics (Figure S1) and annotated secondary structure representations (Figure S2). We note that the expected conformation of the bulge in the context of the full intron likely does not resemble any of the earlier X-ray or NMR structures. Despite sequence differences, the bulge conformation in the full intron will probably be similar to the more recent X-ray structures of the full *Oceanobacillus iheyensis* self-spliced group IIC intron where tertiary interactions stabilize a different bulge conformation (PDBs: 3BWP, 3EOG, 3EOH, and 3IGI) (Toor et al. 2008, 2010). Collectively, the various bulge conformations observed in the earlier NMR and crystal structures, and those we report upon re-refinement, suggest that the bulge structure clearly differs when in an isolated solution environment, is influenced by sequence, crystal packing and tertiary packing, and is likely dynamic. Except for the bulge region, most of the other elements of the D5 structure are quite similar between ai5γ_NMR and PL_NMR. One remaining difference is the overall structural length of D5. The structures of ai5γ_NMR are more extended while those of PL_NMR are compact and similar in length to the x-ray structure of ai5γ-D5. The other difference of significance between the published studies relates to the determination of divalent ion binding as determined by NMR chemical shift perturbations upon addition of MgCl_2_. It was not obvious to us whether real differences in ion binding or structure exist between the RNA constructs, or whether the published observations reflect subtle differences in NMR methods and/or experimental conditions, despite apparently strong similarities in the experiments and refinement protocols.

Together, these two structures and their previously accumulated and published data (chemical shifts, restraints, and chemical shift perturbations) provide an intriguing opportunity to validate the MD simulations, to assess simulation refinement protocols which use explicit solvent and modern force fields, and to ultimately determine whether the significant differences in these structures reported are real or artifacts from the refinement process. We show that re-refinement leads to two very similar structures that appear to better satisfy the NMR data. In addition, beyond the bulge region (which is strongly influenced by packing effects and tertiary interactions), the stem and loop regions around the bulge better match the ai5γ-D5 (PDB: 1KXK) crystal structure than the previously published NMR structures. Yet, the results also suggest that a careful balance between relative weights of the force field compared to the experimental data is required, that the experimental data has to be carefully screened, that there are clear sampling limitations, and that there are still known and emerging force field limitations. Taken together, these observations preclude the use of automation to automatically refine structures. The previous and current success of these methods suggest that some published structures might be improved using explicit solvent MD refinements and that such techniques should be more routinely applied in future structure refinement projects.

## Materials and methods

### Coordinates and restraint data

The coordinates and restraint data for the ai5γ_NMR and PL_NMR structures were retrieved from the RCSB Protein Data Bank website using the PDB codes 1R2P and 2F88, respectively. Of the ten conformers contained in each PDB file, only the first five were used in simulations. The distance restraint data was thoroughly checked for atom naming mismatches between the restraint file and the PDB files. Mismatched restraints were corrected based on visual inspection of the structures (common mismatches included H62 → H61 or H5′1 → H5′2 transpositions). Distance restraints were weighted at 20 kcal/mol-Å within 0.5 Å of the bounds of the flatwell restraint and at 20 kcal/mol-Å^2^ outside this range, unless otherwise noted. The applied dihedral restraint data from the two earlier refinements were consolidated and reconfigured to produce a more consistent and liberal set of dihedral restraints and also to eliminate minor differences in the conventions used by the ai5γ_NMR and PL_NMR authors. Backbone torsions of the helical regions (torsions 1:γ–8:γ, 10:ε–14:γ, 19:ε–23:γ, 27:ε–34:ε) were restrained to A-form values (±15°) while those in the remaining non-canonical regions were left unrestrained. The χ torsion angle was restrained to *syn* (70 ± 30°) for G26 and *anti* (−160 ± 15°) for all others. Sugar puckers were restrained (i.e., torsions δ, ν1, and ν2) in a similar manner as described in the original publications: for ai5γ-D5, A16 and C25 were restrained to C2′-endo, whereas G15, A17, and A18 were unrestrained, and the remaining were restrained to C3′-endo; for PL-D5, A16, A24, and A25 were restrained to C2′-endo, and the remaining were restrained to C3′-endo. Torsion restraints were weighted at 500 kcal/mol-rad within 1° of the bounds of the flatwell restraint and at 500 kcal/mol-rad^2^ outside this range, unless otherwise noted. Relative RDC restraint weighting, set with the “dwt” keyword in AMBER, was chosen to be the highest value that did not cause SHAKE errors during simulation (dwt = 0.02 for ai5γ-D5, dwt = 0.01 for PL-D5). Base pair planarity restraints were not applied. All restraints were converted to the AMBER formats using in-house scripts and scripts available in AmberTools. The restraint files used during the refinement are supplied in the Supporting Information.

### Building, heating, and equilibrating solvated structures

All MD simulations were performed using the AMBER and AmberTools suites of software (Pearlman et al. 1995; Case et al. 2005). The PDB structures were parameterized using the AMBER ff99bsc0 (Perez et al. 2007b) force field, and were solvated in an icosahedral TIP3P (Jorgensen et al. 1983) water box out to at least 10 Å in each direction from the solute followed by net-neutralization with Na^+^ ions and addition of ~200 mM NaCl using the Joung and Cheatham ion parameters (Joung and Cheatham 2008, 2009). The Na^+^ cation was chosen for initial investigations due to the salt crystallization artifacts seen with the earlier K^+^ parameters (Auffinger et al. 2007), despite the fact that K^+^ was used in the NMR buffer. The Na^+^ cation was used throughout this work, albeit with the improved parameters, in order to maintain consistency with our older data. In total, the solvated systems contained between 9,000 and 12,000 residues, corresponding to approximately 29,000–37,000 atoms. After building the coordinate and parameter/topology (prmtop) files, the positions of all the ions in the coordinate files were randomized using ptraj, ensuring that ions were at least 6 Å from an RNA atom and 4 Å from each other. The particle mesh Ewald method (Essmann et al. 1995) was used to handle electrostatic interactions using a 9 Å cutoff with default parameters (including an ~1 Å grid spacing, cubic spline interpolation, and a direct space cutoff tolerance of 0.000001). Lennard-Jones interactions were also treated with a 9 Å cutoff and the pairlist built to 10 Å was automatically rebuilt if any atom moved more than 0.5 Å since the previous update. Each system was first relaxed with 1,000 steps each of steepest descent and conjugate gradient minimization while RNA atom positions were restrained with 25 kcal/mol-Å^2^ positional restraints. Continuing with the same positional restraints, the system was slowly heated from 100 to 300 K over the course of 100 ps at constant volume. After heating, the system was repeatedly minimized (1,000 steps each, steepest descent and conjugate gradient) and equilibrated at constant pressure (for 50 ps each round) using gradually weaker positional restraints (5.0, 4.0, 3.0, 2.0, 1.0, and 0.5 kcal/mol-Å^2^). For restrained simulations, distance and torsion restraints as previously described were enforced during each step of the equilibration process. A final equilibration step for the restrained simulations was included following the 0.5 kcal/mol-Å^2^ position restrained equilibration. This step consisted of a relaxation period of 2 ns at constant pressure without positional restraints and with distance and torsion restraints at 10 % of normal strength.

### Restrained production simulations

Production simulations were performed at constant pressure and temperature using the Berendsen algorithm (Berendsen et al. 1984) for scaling. The heat bath and pressure coupling time constants were set to a loose value of 5 ps. Chemical bonds to hydrogen atoms were constrained using the SHAKE algorithm (Ryckaert et al. 1977; Miyamoto and Kollman 1992), which permitted a time step of 2 fs for production simulations. Translational and rotational center-of-mass motion was removed every 500 steps. Coordinates of the system were recorded every picosecond during simulation. Distance/torsion angle (DA) restrained simulations were performed using the AMBER’s PMEMD program. Restrained simulations using distance/torsion-angle/RDC (DAR) restraints were performed using AMBER’s sander program (PMEMD is generally faster than sander however PMEMD does not yet implement RDC restraints). DAR restrained simulations were not started from an independent equilibration, but rather were started from the final frame of the corresponding DA restrained simulation. To accomplish this, the RDC alignment tensor was first minimized to best fit the RNA structure. Then the DAR simulation was started using the tensor values obtained in the minimization. Every time a DAR simulation was restarted, the alignment tensor values were obtained from the final step of the previous output file and used as the starting values for the next calculation. A complete listing of the simulations is given in Table 1, noting that the five independent runs originated from the first five representative NMR structures from the 1R2P and 2F88 PDB files.

**Table 1:** Simulation details and nomenclature for the various refinements

Name	No. of simulations	Simulation notes	Time^a^	
ai5γ_UR	5	Unrestrained simulation	111	ns
ai5γ_DA	5	Distance and angle restraints enforced	20	ns
ai5γ_DAR	5	Distance, angle, and RDC restraints enforced	8	ns^b^
ai5γ_mDA	5	Modified distance and angle restraints enforced	20	ns
ai5γ_mDAR	5	Modified distance, angle and RDC restraints enforced	8	ns^b^
PL_UR	5	Unrestrained simulation	92	ns
PL_DA	5	Modified distance and angle restraints enforced	20	ns
PL_DAR	5	Distance, angle, and RDC restraints enforced	8	ns^b^
PL_mDA	5	Modified distance and angle restraints enforced; additional heating step	23	ns
PL_mDAR	5	Modified distance, angle, and RDC restraints enforced	11	ns^b^

^a^The time listed is for the minimum trajectory length of the five models

^b^DAR simulations started from the final frame of corresponding DA simulation

The structure refinement protocol presented here significantly extends the procedure used to generate the ai5γ_NMR (Sigel et al. 2004) and PL_NMR (Seetharaman et al. 2006) structures. Specifically, significantly longer MD simulations were performed including explicit solvent and mobile counterions, modern force fields, and proper treatment of the long range electrostatic interactions. Longer simulation, and in some case heating, provided significantly more sampling of potential RNA structure and helped identify where structures may have otherwise been trapped due to previously insurmountable barriers. In contrast, both earlier publications report using CNS to generate an extended structure, followed by the selection of 100 starting structures generated from different random initial velocities. The starting structures were relaxed using high-temperature, torsion-angle dynamics, slow cooling using distance and angle restrained molecular dynamics, and minimization. Total molecular dynamics for each structure in the earlier refinement protocols did not exceed 250 ps. The PL_NMR structures were then further refined based on the RDC data using a more extensive protocol.

### Analysis

All PDB and trajectory structures were visualized using UCSF Chimera (Pettersen et al. 2004). Structure snapshots were also generated using Chimera. RMSD values were generated using AMBER’s ptraj module and results were plotted using Grace or Microsoft Excel. Distance and dihedral measurements were calculated and analyzed using ptraj and in-house scripts. Clustering was performed in ptraj (Shao et al. 2007) using the following settings: average-linkage algorithm, cluster count set to 5, rms similarity metric on base heavy atoms only, and sieve set to 5. To generate representative structures for the restrained simulations, the average structure of the dominate cluster for each trajectory was minimized with full restraints. Grid analysis (Cheatham and Kollman 1997) was performed using ptraj and visualized in Chimera. Occupancy analysis of water and Na^+^ was performed using the hbond command in ptraj.

## Results

### Initial results with unrestrained simulations

Prior to running the restrained simulations, we performed a set of ~100 ns unrestrained simulations using the ai5γ_NMR and PL_NMR starting structures in order to evaluate the AMBER ff99bsc0 force field; a summary of all of the simulations performed is provided in Table 1 and a figure highlighting the structural and sequence differences is shown in Fig. 1. These simulations, named ai5γ_UR and PL_UR, respectively, were built and equilibrated using the same procedure as for the restrained simulations except without any of the steps related to the distance, torsion, or RDC restraints. The initial results from these simulations led us to begin a more thorough investigation using restrained simulations for two reasons. The first is related to the structural compactness of the ai5γ_NMR and PL_NMR structures. One of the more striking differences between the published ai5γ_NMR and PL_NMR structures is the overall structural length, as measured from the top of the tetraloop to the bottom of the lower helix. The PL_NMR structures display a compacted global conformation that is consistent with and almost as compacted as the x-ray structure of ai5γ-D5 (Zhang and Doudna 2002; Seetharaman et al. 2006), whereas the ai5γ_NMR structures are much more extended (Figure S1). In contrast, when we compared the average structures for the unrestrained ai5γ_UR and PL_UR simulations, both sets of structures adopted the more compact conformations. Visual inspection of the ai5γ_UR trajectories revealed that the end-to-end distance of the structures underwent an approximately 15 Å compaction within the first 10 ns of the simulations. This rapid compaction on the MD simulation time scale suggests that the extended structure is not compatible with the force field, a force field as discussed in the introduction that is known to fairly reasonably model many RNA structures. The PL_UR structures, whose starting structures were already more compact, underwent no appreciable compaction during simulation. This likely leads to the rather significant difference in plateau RMSD values between the ai5γ_UR and PL_UR simulations (Figure S3). Note that although the RMSd values plateau and appear relatively small, at least in the case of the PL_UR structures with RMSd values in the ~3–6 Å range, structural disruption in the bulge and loop regions was evident. However, the similarity of the two sets of unrestrained simulation structures after MD simulation led us to wonder if perhaps the conformation of these two molecules were more similar than the conventionally refined structures suggest or if the minimalist gas-phase refinement protocol employed previously was insufficient to refine the structures.

The second reason these simulations encouraged us to perform a more detailed analysis was related to the localized loop and bulge structural features. We found that during both the ai5γ_UR and PL_UR simulations these regions experienced significant structural degradation. For instance, at various times in the five independent simulations the loop conformation would transition to a pathological, yet stable, geometry that often persisted for the rest of the trajectory. It was soon clear that accurate modeling of these molecules could not be achieved using the MD force field alone, and thus we decided to perform restrained simulations using the original NMR data. While the RNA force field parameters have considerably improved (Cornell et al. 1995; Cheatham et al. 1999; Foloppe and MacKerell 2000; Wang et al. 2000; Perez et al. 2007b; Banas et al. 2010; Yildirim et al. 2010; Denning et al. 2011), our results suggest the geometries of the refined structures presented in the current study are primarily determined by the experimental restraints. The explicit solvent environment and updated force field play a secondary yet critical role, as the resulting structures appear to be improved compared to those obtained using conventional methods.

### Restrained simulations produce conformational rearrangements in ai5γ-D5 and PL-D5

**Fig. 2:**
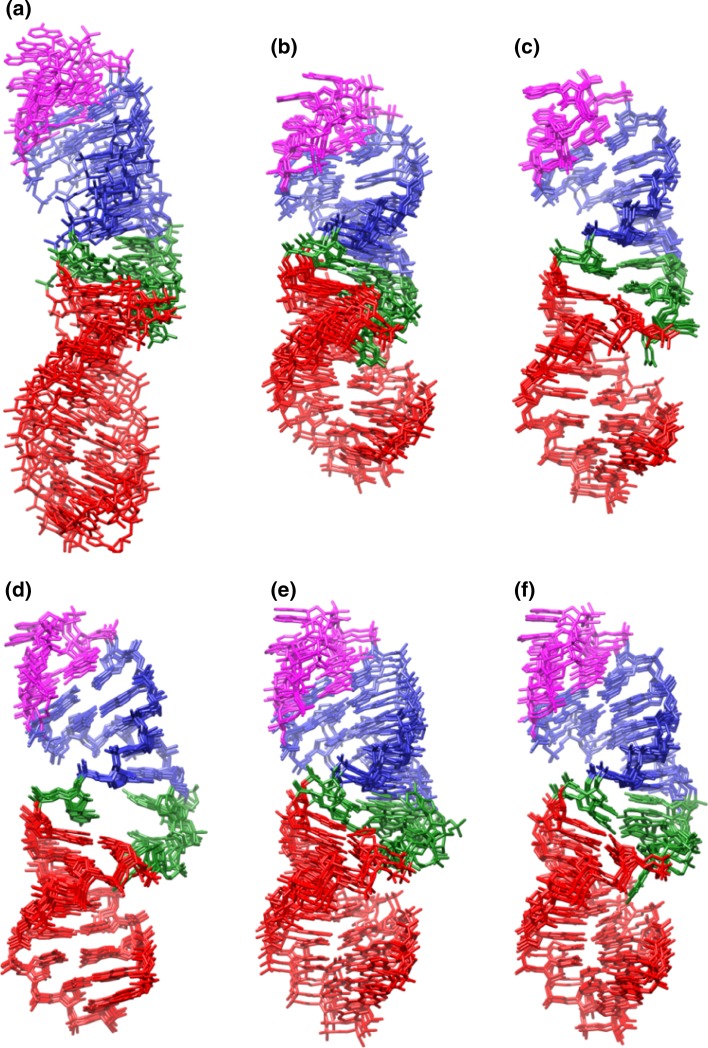
Representative structures for **a** ai5γ_NMR, **b** ai5γ_DAR, **c** ai5γ_mDAR, **d** PL_NMR, **e** PL_DAR, and **f** PL_mDAR. The “NMR” code denotes the original ensemble from earlier refinement (ai5γ_NMR is PDB: 1R2P, PL_NMR is PDB: 2F88). “DAR” refers to the representative structure from the dominant ensemble sampled during the five independent explicit water MD simulations with distance, torsion angle, and residual dipolar coupling restraints enforced. “mDAR” is identical to “DAR” except with small modifications to the restraint list or the equilibration protocol as discussed in the main text. See Table 1 for details

In addition to giving clues about structure compaction, our initial investigation of unrestrained simulations led us to hypothesize that the ai5γ-D5 and PL-D5 structures are more similar than the reported NMR structures suggest. To investigate this possibility we ran simulations with distance, angle, and residual dipolar coupling (RDC) restraints imposed (i.e., ai5γ_DAR and PL_DAR). The resulting trajectories were clustered and a representative structure from the most populated cluster for each trajectory was minimized. These minimized structures (five total, one for each of the five models of a given each simulation type), served together as representative structures for each type of simulation (summarized in Fig. 2 and Table 2). A pairwise heavy atom RMSD measurement between the ai5γ_DAR and PL_DAR structures, which excluded the base atoms for the three differing residues, was much lower (3.33 Å) than the corresponding measurement between the ai5γ_NMR and PL_NMR structures (6.03 Å) (RMSD data listed in Table 3). A significant portion of this decrease was likely due to the structure compaction of ai5γ_DAR.

**Table 2:** Structural statistics for ai5γ-D5 and PL-D5 representative structures

Model	ai5γ-D5			PL-D5		
	NMR	DAR	mDAR	NMR	DAR	mDAR
No. of structures	5	5	5	5	5	5
No. of distance restraints	595	595	592	549	549	543
No. of dihedral restraints	238	238	238	247	247	247
No. of RDC restraints	24	24	24	37	37	37
Avg. RMSd of distance (Å)	0.021	0.020	0.016	0.019	0.028	0.013
Avg. RMSd of dihedral (°)	0.482	0.378	0.365	0.703	0.152	0.239
Avg. RMSd of RDC (Hz)	1.897	2.939	3.067	3.115	4.324	4.390
Avg. RMSd from ideal bonds	0.011	0.011	0.011	0.011	0.011	0.011
Avg. RMSd from ideal angles	2.313	2.426	2.425	2.281	2.462	2.500
No. Distance viol. > 0.2 Å	4	5	0	2	13	0
No. Angle viol > 5.0°	5	4	3	7	0	1
Overall heavy atom RMSD						
Avg. RMSd from mean	2.58 ± 0.84	1.41 ± 0.29	0.66 ± 0.32	0.83 ± 0.12	1.69 ± 0.45	1.14 ± 0.28
Avg. RMSd pairwise	4.09 ± 1.20	2.10 ± 0.87	1.04 ± 0.46	1.30 ± 0.24	2.56 ± 1.04	1.70 ± 0.76

Structures are depicted in Fig. 2. Definitions of NMR, DAR, and mDAR as per Table 1

**Table 3:** Pairwise RMSD measurements

Comparison	Pairwise RMSD
ai5γ_NMR vs. PL_NMR	6.03 ± 1.00
ai5γ_NMR vs. ai5γ_DAR	7.18 ± 1.52
ai5γ_NMR vs. ai5γ_mDAR	5.61 ± 1.22
ai5γ_NMR vs. PL_DAR	6.55 ± 1.69
ai5γ_NMR vs. PL_mDAR	6.18 ± 1.34
PL_NMR vs. ai5γ_DAR	4.29 ± 0.52
PL_NMR vs. ai5γ_mDAR	2.71 ± 0.15
PL_NMR vs. PL_DAR	2.67 ± 0.63
PL_NMR vs. PL_mDAR	2.21 ± 0.40
ai5γ_DAR vs. PL_DAR	3.33 ± 0.61
ai5γ_DAR vs. PL_mDAR	3.23 ± 0.70
ai5γ_mDAR vs. PL_DAR	2.33 ± 0.82
ai5γ_mDAR vs. PL_mDAR	1.95 ± 0.40
ai5γ_DAR vs. ai5γ_mDAR	3.13 ± 0.66
PL_DAR vs. PL_mDAR	2.00 ± 0.88

Definitions as per Table 1

**Fig. 3:**
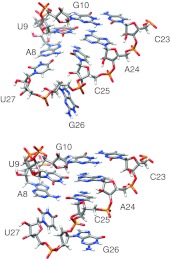
The bulge structures from the re-refined NMR structures: ai5γ_DAR (*top*) and ai5γ_mDAR (*bottom*)

**Fig. 4:**
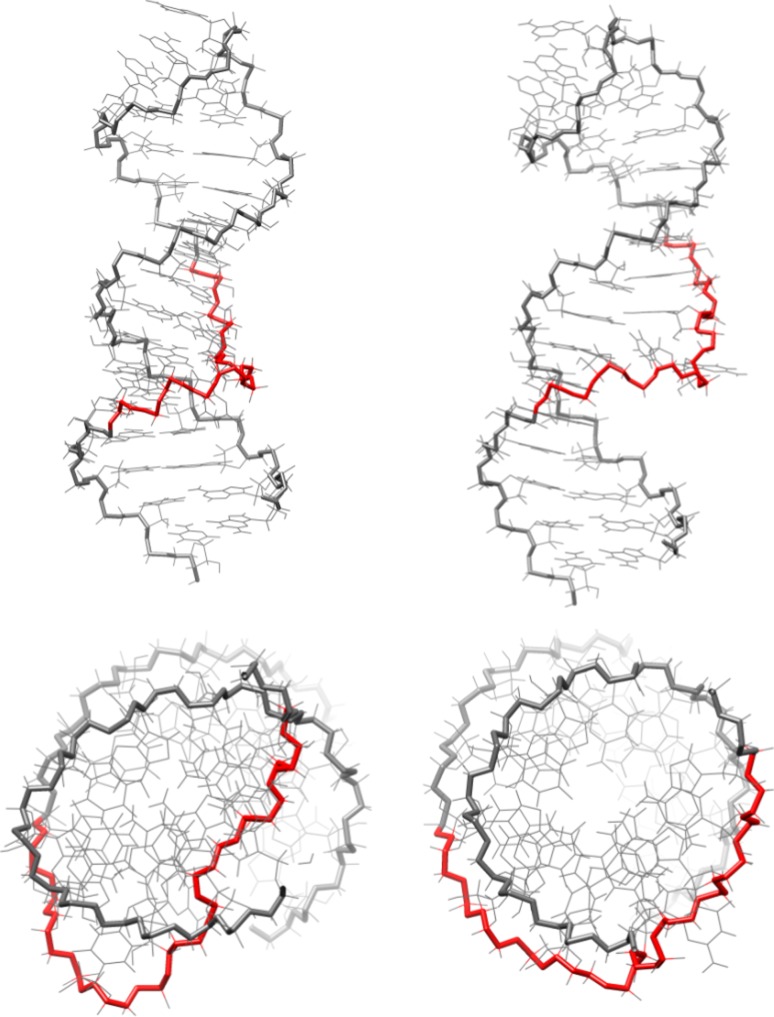
Side and top views of ai5γ_DAR (*left*) and ai5γ_mDAR (*right*). The top view has been truncated to focus on the bulge region. The backbone near the kink region has been highlighted *red*

In addition to a lower inter-structure pairwise RMSD with PL_DAR, ai5γ_DAR also has a much lower intra-structure pairwise RMSD (2.10 Å) as compared to the original ai5γ_NMR structures (4.09 Å). Other than global compaction of the structure, the most significant differences between the ai5γ_NMR and ai5γ_DAR structures occur in the bulge region. In four out of the first five ai5γ_NMR structures, U9 is positioned below residues 24 and 25. For ai5γ_DAR, U9 is directly adjacent to A24 and appears to form Watson–Crick bonding in three of the five structures. The other two structures show U9 disengaged from A24 while still maintaining an adjacent stance. In all five ai5γ_DAR structures, C25 is pressed against the major groove side of the A8-U27 base pair, forming a hydrogen bond between A8 H62 and C25 N3 (Fig. 3, top). This hydrogen bond seems unlikely due to the high angle between the base plane of A8 and C25, and is probably exaggerated by the force field which allows such bonding at any angle. The lower position of C25 in the ai5γ_DAR structures is likely caused by another interesting feature of the bulge region. The kink structure of backbone residues A24–U27 is even more pronounced in the simulation structures than is observed in the NMR structures. The otherwise smooth curve of the backbone is disrupted between G26 and U27, forming a near right-turn in the helix when viewed from above (Fig. 4, orange). In the NMR structures, the kink maintains an upward direction throughout the bulge region. In contrast, the ai5γ_DAR kink briefly travels downward as it cuts across the major groove and forces C25 into a very low position. The orientation of G26 also differs somewhat between the simulation and NMR structures. In most of the five ai5γ_NMR structures (models 1-5 from the PDB file), the base plane of G26 is close to perpendicular with the vertical axis of the lower helix. In contrast, most of the ai5γ_DAR structures have the base plane parallel to the vertical axis of the lower helix.

The differences between the PL_NMR structures and PL_DAR structures are not as drastic as those for ai5γ. In contrast to the results for ai5γ, the intra-structure pairwise RMSD is higher for PL_DAR (2.56 Å) than for the PL_NMR (1.30 Å). One of the most obvious differences between the PL_DAR and PL_NMR structures is that during equilibration and relaxation, residue A25 of model 4 shifted from a partially extruded position in the minor groove to a stacked position within the helix. Thus the PL_DAR ensemble has three of five structures with A25 stacked, whereas the PL_NMR structures have two of five (ignoring structures 6–10 in the published PDB structure file). All five representative structures for PL_DAR show U9 participating in a hydrogen bond with either A24, A25 or G26. In the two structures with A25 extruded, U9 interacts with A24. Of the three structures with A25 stacked in the helix, two show U9 interacting with A25 and one shows a hydrogen bond between U9 H3 and G26 N7. During simulations of the former case, U9 shifts back and forth between interactions with A24 and A25. In the latter, the U9 H3–G26 N7 hydrogen bond is particularly stable throughout the trajectory, leaving A24 and A25 stacked above U9. In all five simulations, these interactions close the “hole” described by Seetharaman et al. in reference to the PL_NMR structures. Interestingly, for the three structures with A25 in the stacked position, G26 no longer “packs into the major groove against G8” (as in all five PL_NMR structures), but points away from the major groove with the base plane parallel with that of U9. For the two structures with A25 extruded into the minor groove, G26 remains oriented towards the major groove.

### Troubleshooting problematic or unusual regions in the refined ai5γ-D5 and PL-D5 structures

On closer inspection of the refined models, some features of both the ai5γ_DAR and PL_DAR structures seemed problematic. For ai5γ_DAR, there were three distance restraints in the bulge region with consistently large upper bound violations during simulation (these restraints connected the following atoms: U7 H1′-A28 H2, G26 H8-A28 H1′, G26 H8-U7 H3). Two of these involved the atom G26 H8. On closer inspection it seemed possible that these two restraints were responsible for the severe kink in the backbone noted by the authors of ai5γ_NMR structures as being a very unusual conformation. Given the high upper and lower bounds of these restraints, one of the corresponding authors (Samuel Butcher, personal communication) suggested to us that these restraints were derived from weak NOEs that may be mediated by spin diffusion. Even though the U7 H1′–A28 H2 restraint violation was probably a side effect of the other two restraints, we decided to investigate the effect of removing all three problematic restraints. Identical simulations to ai5γ_DAR were then run with the three aforementioned restraints removed to generate the simulations that are designated as ai5γ_mDAR.

Additionally, the unrestrained simulations suggested that the positioning of the A25 residue in the PL_DAR structures might also be problematic. Although the unrestrained simulations are imperfect due to force field deficiencies, we never observed A25 extruded into the minor groove during the 100 ns of unrestrained simulations. As mentioned previously, we also found that one fully restrained simulation showed A25 move from the extruded position to the stacked position and we therefore considered whether this conformation might be preferred. To investigate this, we tested several different equilibration and relaxation conditions before choosing a procedure for the modified restrained simulations. Many of the conditions resulted in either one or two of the three extruded structures transitioning to a stacked structure. Critically, in none of these conditions did A25 transition from a stacked conformation to an extruded conformation. In one condition, all three of the extruded structures transitioned to stacked structures. This condition involved three alterations to the PL_DAR simulation procedure: (1) the removal of the Watson–Crick base pair restraints, but not the NOESY restraints, between residues G8 and C27 (which are not present for A8/U27 in ai5γ_DAR), (2) increasing the weight of distance restraints from 20 to 50 kcal/mol as well as increasing the G26 χ dihedral restraint from 500 kcal/rad to 1,000 kcal/rad, and (3) heating to 700 K with restraints at 8 % strength to allow structural relaxation, followed by a smooth increase of restraint weight to 100 % strength prior to the production simulation. The rationale for removing the Watson–Crick base pair restraints was to allow structural transitions in the bulge region that may otherwise be hindered. The changes to the restraint weighting were made to ensure the enforcement of distance restraints and prevent G26 from flipping to the *anti* conformation (a frequent problem for previous attempts) while the heating period was intended to enhance conformational sampling. These simulations were named PL_mDAR.

### Analysis of the bulge and loop regions in optimized, restrained simulations

The removal of the three problematic restraints from the ai5γ restraint list makes a significant difference in the bulge region of the ai5γ_mDAR structures. First, the intra-structure pairwise RMSD of the ai5γ_mDAR structures (1.04 Å) is significantly lower than ai5γ_DAR (2.10 Å) (Table 2). The sharp kink observed in the bulge of the ai5γ_DAR structures is replaced by a smooth, upward trending backbone in the ai5γ_mDAR structures (Fig. 4, green). Rather than being drawn below the major groove face of the A8-U27 base pair, C25 is positioned above A8-U27 and its base plane is parallel with A8′s in the ai5γ_mDAR structures (Fig. 3, bottom). This positioning puts U9 directly in between the A24 and C25 bases and during simulation U9 alternates hydrogen bonding between A24 and C25. Occasionally, C25 N3 or O2 also form a hydrogen bond with the A8 H61, H62 atoms. In contrast to the ai5γ_DAR structures, four of the five ai5γ_mDAR have the base plane of G26 perpendicular to the helical axis, thus pointing away from the major groove.

**Fig. 5:**
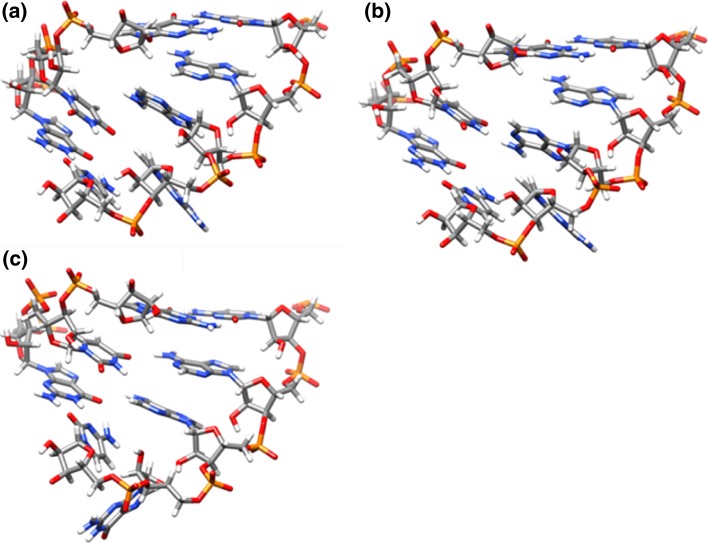
The three bulge conformations adopted by PL_mDAR

For the PL_mDAR structures, the removal of the A8-U27 Watson–Crick base pair restraints and subsequent heating yielded representative structures with a lower pairwise RMSD value (1.70 Å) than that of the PL_DAR structures (2.56 Å), although not as low as the original NMR structures (1.30 Å). During the five PL_mDAR simulations, three different bulge motifs dominate, all of which are more packed than the open bulge observed in the original PL_NMR structures. In Model 1, the U9H3-G26N7 hydrogen bond forms (Fig. 5a). As was the case for the same hydrogen bond seen before in model 2 of PL_DAR, this structure is quite stable during the simulation. Models 2 and 3 show U9 interacting with A25, yet U9 never quite reaches A24 (Fig. 5b). This conformation is more dynamic, with U9 oscillating above and below A25. Finally, models 4 and 5 show U9 placed between A24 and A25 (Fig. 5c). In these simulations, U9 alternates between interactions with A24, A25 and a shared interaction occurs between the two. Interestingly, the positioning of U9 is correlated with the positioning of G26. In models 1-3, where U9 is interacting with A25 or A26 (but not A24), G26 remains partially in the helical stack with its base plane perpendicular to the helical axis. However in models 4-5, with U9 interacting with A24 and A25, G26 is pushed out of the helix and the base plane is parallel to the helical axis. Contrary to the inferences from the ai5γ_NMR and PL_NMR structures, the ai5γ_mDAR and PL_mDAR structures are quite similar to each other. The average heavy atom pairwise RMSD (excluding the base atoms of differing residues) is much lower (1.95 Å) than the first simulation structures (3.33 Å) and the published NMR structures (6.03 Å). In addition, the lower helix regions of the re-refined structures are very similar to each other and better match the earlier crystal structure (PDB: 1KXK). The average heavy atom pairwise RMSD to the crystal for the lower helix is 2.36 Å and 1.50 Å for the original NMR structures and 0.81 and 1.13 Å for the re-refined structures (for ai5γ and PL, respectively). The remaining structural differences in the bulge are likely related to the accommodation of the larger A25 in PL-D5 as opposed to C25 in ai5γ-D5.

The other non-canonical structure of interest, the GAAA tetraloop, is reasonably similar between the NMR structures and among each of the restrained simulation structures. These structures are also closer to the earlier crystal structure. Whereas the RMSD values for residues 10–23 are 1.64 and 1.79 Å when the original NMR structures are compared to the crystal, the re-refined structures yield values of 1.37 and 0.92 Å (for ai5γ and PL, respectively). However, some variation in the backbone torsions are observed, such as that of the γ torsion of residue A16, which we attribute to the bsc0 modifications of the AMBER ff99 parameters which disfavor γ in the *trans* configuration (Perez et al. 2007b). In both the ai5γ_NMR and PL_NMR structures, γ is in the *trans* configuration while in each of the ai5γ_mDAR and PL_mDAR structures this torsion is in the *gauche*+ position. In addition to the backbone differences, the base orientations in the tetraloop were compared between the NMR structures and the restrained simulation structures. Previous work by Correll and Swinger (2003) identified aspects of the GNRA tetraloop that commonly vary between one of two conformations. The first of these involves the planarity of the “NRA” portion of the tetraloop with respect to the underlying base pair of the upper helix (i.e., the planarity of residues 16–18 with respect to the base pair formed by residues 14 and 19 in the case of D5). When the NRA bases are planar with the underlying base pair, the conformation is referred to as the “standard orientation”. If the NRA bases depart from planarity, typically tilting upward and away from the underlying base pair, the conformation is named the “altered orientation”. In both cases, the three NRA bases remain stacked together. Visual inspection of both the NMR structures and representative structures from our restrained simulations reveal that nearly all of the tetraloops adopt the altered orientation. This contrasts with results from unrestrained simulations in which the standard orientation seems to be favored.

**Fig. 6:**
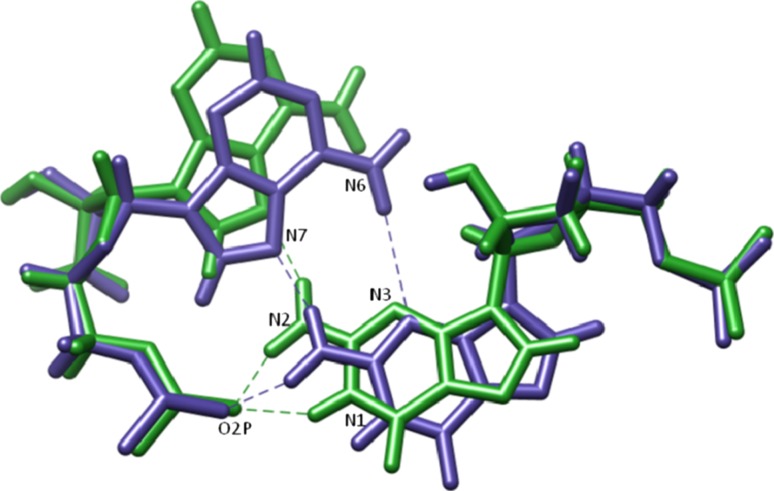
A comparison of the outward orientation (*green*) and inward orientation (*blue*) of the G15–A18 basepair in the GAAA tetraloop

The second feature of interest identified by Correll and Swinger in GNRA tetraloops is the hydrogen bonding network of the first and fourth residues in the tetraloop (i.e., G15 and A18 for D5). In the first case (which we name the “outward orientation”), G15 N2 forms a bifurcated hydrogen bond between both A18 N7 and A18 O2P, while G15 N1 also hydrogen bonds with A18 O2P. In the second case (the “inward orientation”), G15 and A18 shift slightly relative to the backbone which allows for the same bifurcated hydrogen bond between G15 N2–A18 N7/A18 O2P as well as a hydrogen bond between G15 N3 and A18 N6. Interestingly, the ai5γ_mDAR structures seem to fit the outward orientation, whereas the PL_mDAR structures fit the inward orientation (Fig. 6). Both the ai5γ_NMR and PL_NMR structures also appear to adopt the inward orientation although the refinement did not seem to capture the fine detail of hydrogen bonds that stabilize the structure. For instance, in many of the submitted models for both NMR structures, the orientation of G15 N2 does not indicate a hydrogen bond is formed with A18 N7, although the atoms are close in space. The conformational differences found in the loop regions when comparing the ai5γ_mDAR and PL_mDAR structures are surprising given that the NMR conditions are similar and the upper helix and tetraloop sequence is identical. Slight differences in the restraint data likely lead to the observed differences, and as discussed below these conformational differences result in slightly different hydration and Na^+^ binding features. As both loop conformations are observed, and each is consistent with experimental data, it is not possible to directly ascertain which conformation is preferred and/or if the loops interconvert between the two conformations, although likely the two conformations are close in energy which suggests population of both.

### Deviations from ideal geometry when using the AMBER force field

One problem which might deter researchers from using the AMBER force field is the slight deviations of covalent bond angles from ideal geometries. Deviations outside of the expected covalent bond angle range were observed for all of our AMBER refined structures as well as other recent structures (Paulsen et al. 2010b; Tolbert et al. 2010) when submitted to the ADIT-NMR (AutoDeposit Input Tool for NMR structures: http://deposit.bmrb.wisc.edu/bmrb-adit/) from the RCSB website (http://www.rcsb.org). The origin of the deviations results from the use of shared, transferable and rather generic atom types for the nucleobases (such as CT for all tetrahedral carbons and OS for O2′, O3′, O4′, and O5′) rather than specific types for each different atom to more accurately represent nucleoside geometry. Although the deviations (on the order of ~5° or less) are outside the range observed in experimental databases (Clowney et al. 1996; Gelbin et al. 1996), these deviations are within the range of thermal fluctuation and likely have a small impact on the overall structural quality due to compensation by the many degrees of freedom in large biomolecules. Addressing these deviations will require an overhaul of the atom type naming system used by the AMBER force field and is the subject of ongoing research. In addition to the deviations observed for covalent bond angles, some deviations were also observed in base planarity (on the order of 0.1 Å rmsd or less), which likely reflect restraint strain on the relatively soft improper torsion parameters used in the AMBER force field to maintain planarity.

### Comparision of representative ensembles with and without RDC restraints

A comparison between the simulations run with and without RDC restraints (DAR and DA, respectively), suggests that the RDCs primarily affect the structural compactness of these RNA structures, but not the local conformations. Both the ai5γ_mDAR and PL_mDAR simulations resulted in an increase of overall structural length (measured by the distance between A16 P and C34 P). The average structure length for ai5γ_mDA and ai5γ_mDAR, taken from representative structures, was 47.0 and 54.5 Å, respectively. The increase was less dramatic for PL, for which the average structure length was 52.8 and 53.4 Å for PL_mDA and PL_mDAR, respectively.

These results are consistent with a recent study by Tolbert et al. (2010) of a purely A-form RNA double helix, which suggested that a wide range of A-form structures are accessible when using only distance and torsion restraints. In contrast to what was observed by Tolbert et al., the addition of orientational restraints resulted in structural expansion, not contraction. However, the differences between the simulations (pure helical RNA vs. non-canonical RNA, implicit solvent vs. explicit solvent) preclude further conclusions.

### Solvation and Na^+^ density during simulation

In addition to structural information, the publications describing both the ai5γ_NMR and PL_NMR solution structures also included data describing chemical shift perturbations observed upon titration of D5 RNA with Mg^2+^. The results for ai5γ_NMR, which only tracked 1D proton shift changes, differ somewhat from those for PL_NMR, which included more detailed 2D ^1^H–^13^C and ^1^H–^15^N chemical shift data. However, these differences could be attributed to either a real difference in structure, simply a difference in the experimental techniques, or perhaps a combination of these differences. A detailed examination of Na^+^ position during the simulation provided a plausible explanation for the results observed in the Mg^2+^ binding experiments. Our simulations were performed with monovalent salt due to the lack of good parameters for divalent cations, the absence of polarization, and conformational sampling limitations. Although the replacement of Mg^2+^ with monovalent salt can destabilize RNA, for small RNAs high monovalent salt concentrations are generally a good substitute for physiological Mg^2+^, and typically provide similar structures (Draper 2004; Draper et al. 2005). We studied Na^+^ and water binding during simulation using two techniques. First we generated density grids to map positions of highest density over the course of an entire set of simulations. Second we probed the occupancy of Na^+^ and water within a defined radius for atoms of interest.

**Fig. 7:**
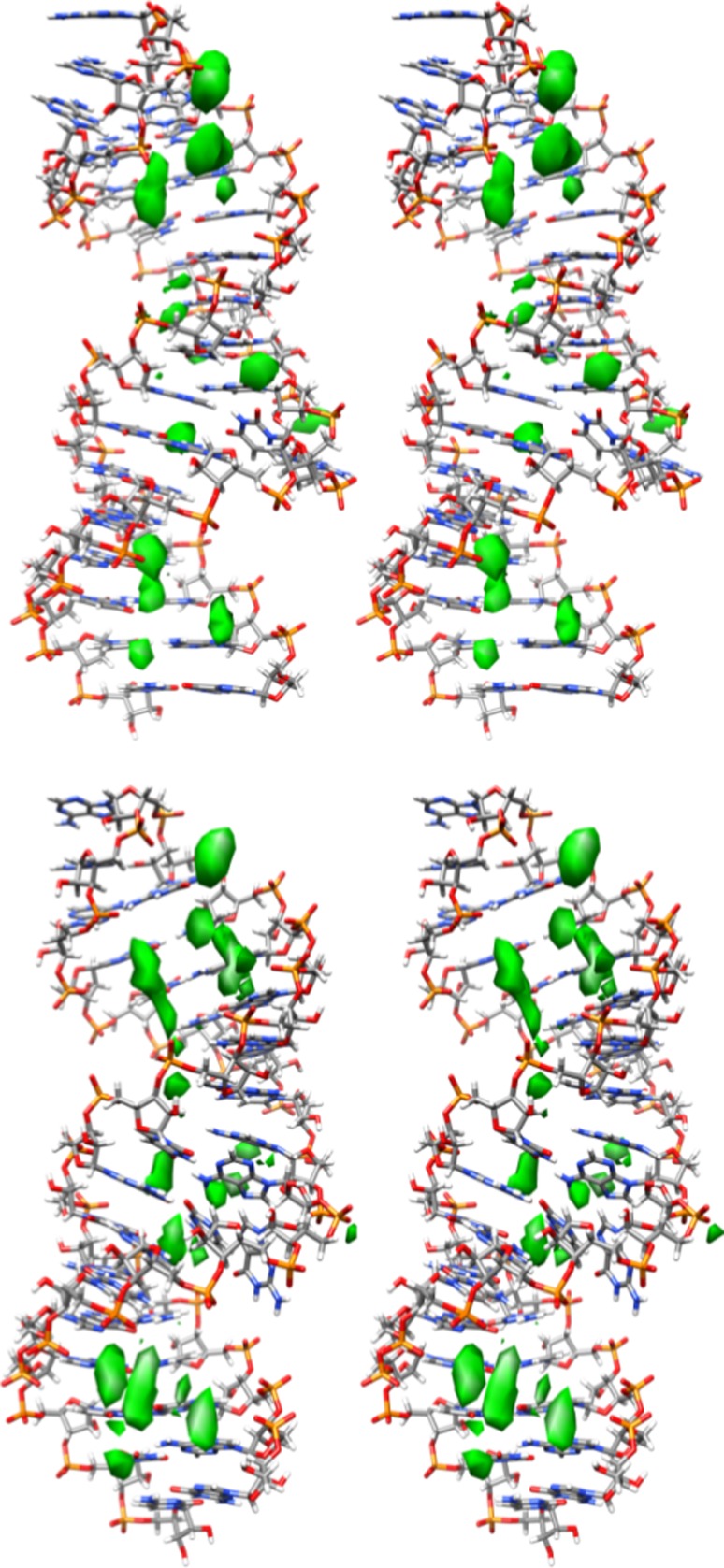
Stereo views of the Na^+^ density grid maps for ai5γ_mDAR (*top*) and PL_mDAR (*bottom*). The isosurface grid density was chosen to show regions of ion localization which were higher than background levels

**Fig. 8:**
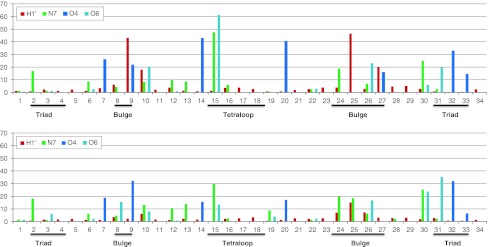
Percent occupation of Na^+^ near selected atoms for all bases. Data for ai5γ_mDAR on top, PL_mDAR on bottom. The cutoff for H1′ was 5 Å. The cutoff for N7, O4, and O6 was 2.8 Å

One of the more significant differences between the results for ai5γ_NMR and PL_NMR involved the triad region (A2, G3, and C4). For ai5γ_NMR, Sigel et al. (2004) found that none of the protons in this region experienced a significant perturbation. However, Seetharaman et al. (2006) reported that N7 of A2, G30, and G31 were significantly perturbed while N7 of G3 was not. A comparison of the 3D grid structures for ai5γ_mDAR and PL_mDAR suggest that Na^+^ binding occurs in the tetraloop, bulge, and AGC triad region for both structures (Fig. 7). This data supports Seetharaman et al. (2006) who suggest that probing for perturbations in the N7 atoms of ai5γ-D5 would also uncover these results. Furthermore, detailed analysis of Na^+^ and water density near each of the triad region base pairs suggests a possible explanation for why N7 of G3 was not perturbed while N7 of A2, G30 and G31 were perturbed (Seetharaman et al. 2006). In the case of A2 N7 and G30 N7, a high density region of Na^+^ is positioned directly off the N7 atom (Figure S4). Occupancy analysis of Na^+^ within 2.8 Å of these two atoms show that for both ai5γ_mDAR and PL_mDAR they rank among the highest in Na^+^ binding out of all N7 atoms (Fig. 8). Although G31 N7 does not directly bind Na^+^, a large region of density exists nearby and the neighboring O6 atom does bind Na^+^ at a high occupancy and probably contributes to the chemical shift. In the case of residue G3, neither N7 nor O6 have a high Na^+^ occupancy, and grid analysis reveals two areas of high water density that form a barrier between the Na^+^ density and residue G3 (Figure S4).

Another region of interest is the bulge (residues 9, 24, 25, and 26; residues 8 and 27 can be included as well). Chemical shift perturbation analysis showed that H1′ protons in residues 9 and 25 of ai5γ_NMR were greatly affected by Mg^2+^ titration, whereas only the aromatic carbon and nitrogen atoms in residue 24 of PL_NMR were affected. As was observed for the triad region, we suspect that carbon and nitrogen chemical shifts in residue 24 would have also been perturbed in ai5γ_NMR if they had been monitored. However, the shifts of C1′ in residues 9 and 25 of PL_NMR were not affected by Mg^2+^ as was seen for the analogous H1′ for ai5γ_NMR. The simulation data clearly accounts for this difference. Visual inspection of the Na + density grid for ai5γ_mDAR reveals a large, high density region straddling the minor groove surface of residues 9, 25 and 27 (Figure S5, top). Na^+^ ions in this region are likely stabilized by the O2 atom of U9, C25, and U27. The positioning of this high density region is therefore quite close to the H1′ atoms of these same residues and occupancy analysis using a 5 Å cutoff reveals that these H1′ atoms are among the nearest to Na^+^ during simulation (Fig. 8). In contrast, no such high density region is observed for PL_mDAR (Figure S5, bottom), and the H1′ atoms of U9, A25, and C27 are not near Na^+^ during the simulation (Fig. 8). It is likely that Na^+^ binding in this region of PL-D5 is not supported for two reasons: (1) the occurrence of adenine at residue 25, rather than cytosine in ai5γ-D5, places a hydrogen atom in the binding region, rather than an oxygen atom, and therefore does not support metal binding and (2) replacement of the A8-U27 base pair in ai5γ-D5 with the G8-C27 base for PL-D5 produces a stronger base pair and does not allow the twisting conformation that permits ai5γ-D5′s U27 O2 to interact with a Na^+^ atom.

**Fig. 9:**
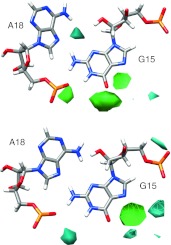
Water and Na^+^ density grid in the region near the non-Watson–Crick G-A base pair of ai5γ_mDAR (*top*) and PL_mDAR (*bottom*)

The tetraloop region is the third area of high Na^+^ binding that is identified using grid analysis (Figure S6). As noted earlier, the ai5γ_mDAR and PL_mDAR loop structures adopt slightly different conformations (both adopt the altered orientation of the base planes, but ai5γ_mDAR displays the outward orientation of G15 and A18, whereas PL_mDAR adopts the inward orientation). A close inspection of the water and Na^+^ density in the tetraloop region reveals that these subtle differences in loop geometry lead to differences in ion binding and solvation (Fig. 9). The solvation patterns from simulation closely match those observed in crystal structures by Correll and Swinger (2003). In the case of ai5γ_mDAR, which adopts the outward orientation, a high density region of water between G15 N3 and A18 N6 likely mediates a hydrogen bonding network (Fig. 9, top). For PL_mDAR, the inward orientation of G15 and A18 exclude this region of solvation, but a new interaction occurs wherein a water molecule mediates a hydrogen bonding network between G15 N1 and A18 O2P (Fig. 9, bottom). The change in base orientation of the tetraloop also results in a shift in the uppermost region of Na^+^ density. For ai5γ_mDAR, the Na^+^ density is situated between N7 and O6 of G15 and lies directly below A17O2P, which appears to be coordinating the ion (Figure S6, top). For PL_mDAR, the Na^+^ is also located between N7 and O6 of G15, but A17 O2P is more distant and any possible coordination interaction is intervened by a region of water density (Figure S6, bottom). In this case, the highly ordered water density that forms around the Na^+^ density is also very similar to that seen in crystal structures, although the ion presence was not reported (Correll and Swinger 2003).

The large simulated Na^+^ density near G15 explains the Mg^2+^ induced chemical shifts for G15 in both ai5γ_NMR (Sigel et al. 2004) and PL_NMR (Seetharaman et al. 2006) and its location in the major groove is consistent with previous NMR work with cobalt(III) hexamine (Rudisser and Tinoco 2000)). It is less clear why A16–G19 C1′ shifts are so heavily affected in PL_NMR, whereas only the H1′ of G15 is affected for ai5γ_NMR. For A16–A19, inspection of the 2D ^1^H–^13^C spectra for H1′–C1′ reveals that the majority of the chemical shift occurs in the carbon dimension (Supplementary Figure 2 in Seetharaman et al. (2006)) and thus would not be revealed in the ai5γ_NMR results where only ^1^H shifts were measured. However, a significant shift does occur in the proton dimension of the PL_NMR G19 H1′–C1′ cross peak, while G19 H1′ of ai5γ_NMR is unaffected by Mg^2+^. Another puzzle is why so many atoms of the tetraloop region have large chemical shift perturbations (according to the PL_NMR results) when the ion binding appears to be limited to the major groove according to the simulation Na^+^ density grid results. Inspection of the medium density solvation shell around the tetraloop region reveals that the base atoms are much more exposed to the bulk solvent than base atoms in the rest of the RNA molecule (data not shown). We propose that this lack of solvent shielding may explain the large Mg^2+^ induced chemical shift perturbations in the tetraloop.

### Simulated annealing with the mDAR restraint sets

To compare the results of our explicit solvent refinement with traditional refinement methods, we performed simulated annealing on the completely extended ai5γ and PL RNA, both in vacuo and with Generalized-Born (GB) implicit solvent, using the mDAR restraint sets. One notable feature of in vacuo results is the presence of sharp kinks near the bulge for both the ai5γ and PL ensembles. Given that these kinks are not observed in the GB ensembles or the explicitly solvated ensembles, these results suggest that in regions with sparse distance restraints, such as the bulge, the lack of a solvation environment can lead to anomalous conformations. Other than the kinked bulge conformation in the in vacuo ensemble, the local conformation of both simulated annealing ensembles are very similar to the explicity solvated ensemble. In contrast, comparison of the average structural length of the GB ensembles reveals that they are somewhat more extended than the explicit ensembles: for ai5γ_mDAR the average structural lengths are 56.9 and 54.5 Å for the GB and explicit, respectively; for PL_mDAR the average lengths are 58.2 and 53.4 Å. In contrast to what was observed for the explicit ensembles, the pairwise RMSD was higher for ai5γ_mDAR GB ensemble (2.54 Å) than that of the PL_mDAR GB ensemble (0.74 Å). The reasons for these differences likely lie with the representative structure selection method. Whereas the explicit solvation representative structures were chosen by their proximity to the centroid of the major cluster during a long simulation, the simulated annealing structures are simply the lowest energy structures which satisfy restraints from a few hundred simulated annealing cycles. It is possible that performing 10,000 or 20,000 simulated annealing cycles (a similar quantity to the frame count in the explicitly solvated simulations) would produce representative structures that are in better agreement with the explicit results. However this has yet to be investigated.

## Discussion

The results presented in this study are immediately relevant to research in experimental structure determination and, more specifically, to refinement of RNA structure from NMR data. First, for structure refinement projects, we find that currently available MD tools with modern simulation protocols, force fields, inclusion of water and mobile counterions, and longer molecular dynamics simulations, offer robust environments for probing structural features that may not be adequately modeled by older and more conventional structure refinement techniques. Our restrained simulations of ai5γ-D5 and PL-D5 produced a set of refined structures that differed significantly from the previously published NMR structures and offer new insights into the similarities and differences of these RNA molecules. For instance, the simulation refined ai5γ_mDAR structures are much more compact than the original NMR structures and more closely resemble both the NMR and simulation structures of PL-D5. Moreover, for regions outside the bulge region (which is strongly influenced by sequence, packing and tertiary interactions) the re-refined structures better match the ai5γ-D5 crystal structure (PDB: 1KXK). We also were able to identify and troubleshoot potentially incorrect regional conformations in the conventionally refined structures of both molecules. For instance, we uncovered three problematic long range distance restraints in the ai5γ-D5 bulge, which when removed generated a smoother backbone trajectory in the bulge region, lower RMSD values, and fewer restraint violations. We also found that in three of the five PL-D5 structures, residue 25 was apparently trapped in a partially extruded conformation that upon heat annealing converted to the stacked conformation having lower RMSD values and fewer restraint violations. Noticing the problematic regions required MD simulations orders of magnitude longer than were previously and typically applied in order to highlight trapped or metastable conformations. Finally, the pairwise RMSD values for the simulation ai5γ_mDAR structures are lower than those for PL_mDAR, which is the reverse of what was found using conventional structure refinement. This is likely due to the greater flexibility in the bulge region of PL-D5 observed during the significantly longer and less-tightly restrained simulations used to re-refine the structure. The need to carefully evaluate the choice of NOE and restraint assignments (to not only check for misnaming, incorrect assignments, and/or potentially anomalous spin diffusion)—coupled with the potential for conformational trapping—suggest that automated NMR refinement of RNA structures remains a challenge. Our results suggest that the refinement of RNA structures requires a careful balance between the strength of the experimental restraints and the influence of the force field, and perhaps most importantly, the resulting structures require careful validation and assessment.

The publication of a self-spliced group IIc intron crystal structure (Toor et al. 2008) reveals that the D5 bulge adopts a different conformation in the context of the entire intron than in isolation. Therefore, while our refinement of the original NMR structures provides new insight into the isolated RNA hairpins, the functional relevance of these structures in the context of the intact intron remains unclear. However, the results clearly suggest that the bulge conformation is sensitive to the surroundings and sequence, and that the re-refinement leads to structures that are more consistent with the available crystal structures. Our simulations also suggest that these hairpin structures, which differ at only three positions, are much more similar than the older conventional refinement techniques indicated. These results also improve our understanding of how differences in primary sequence affect 3D structure, and provide insight into conformational flexibility, solvation, and ion binding. The isolated D5 bulge apparently samples a range of conformations, while tertiary interactions in the context of the entire intron select and stabilize a flipped out conformation necessary to assemble a catalytically active intron (Toor et al. 2008). MD simulations with explicit solvent and updated force fields can potentially uncover minor conformers that nevertheless have functional relevance. The flipped out conformation need not be the lowest energy structure observed by NMR, merely accessible with sufficient frequency to be captured by tertiary contacts in the intron. Accurate “ground state” structures determined from NMR data and explicit solvation MD provide a starting point from which to investigate the dynamical behavior of RNA. The methods employed in this paper should also aid researchers who use structural databases to further refine their models.

Remaining unanswered questions relate to potential disorder and/or dynamics in the bulge and loop regions. MD simulations without experimentally derived restraints are unable to maintain the expected structure; given this, movement away from the experimental structure does not represent true dynamics, but suggests deficiencies in the force field. Researchers may be interested in the minimal set of restraints required to maintain experimentally valid structures. We propose that the minimal restraint requirements for maintaining accurate structure using the AMBER force field would include as many distance restraints in non-canonical regions as possible and orientational restraints such as RDCs to maintain proper structural compaction. Although structures consistent with the experimental data can be maintained via the application of restraints, such restraints will tend to inhibit conformational transitions and dynamics. Given this, it is unclear if the representative structures found in re-refinement completely represent the ensemble of frequently accessed conformations or simply represent the lowest energy structures without a proper depiction of the true disorder or dynamics sampled at room temperature. For instance, depending on the choice of experimental restraints, two GAAA tetraloop conformations, each with distinct solvation and ion binding properties were observed. Both are consistent with experiment, however as exchange was not observed between “outward” and “inward” structures during the re-refinement, speculation on the relative populations or conformational dynamics are not possible. On the other hand, given that both are observed experimentally and are likely nearly iso-energetic, it is likely that both are populated and in dynamic equilibrium. To further resolve these questions through simulation will require improvements in the underlying nucleic acid force fields.

The AMBER force field is continually being developed and refined to produce improved simulation results. Generally these improvements are evaluated in the context of unrestrained simulations. However, evaluating force field performance is difficult given the huge diversity in RNA structure. Our unpublished work suggests that canonical A-form RNA is relatively stable in unrestrained simulations for long periods of time. In contrast, non-canonical regions frequently populate conformations which are not observed in experimental structures suggesting a force field problem. One notable RNA motif for which this occurs is the UUCG tetraloop. Progress towards improving RNA torsional parameters is underway, including recent force field modifications that improve the glycosidic χ in RNA (Banas et al. 2010; Yildirimet al. 2010; Zgarbova et al. 2011).

## Supplementary data

Supplementary data to this article can be found online. This includes a ZIP file containing all of the restraint data files in AMBER format used in this work along with PDB files of the final structures. In addition, a PDF file is supplied that provides six supplementary figures S1-S6. PDB codes for the coordinates are 2LPS and 2LPT, BMRB accession numbers are 18274 and 18275.

## Electronic supplementary material

Below is the link to the electronic supplementary material.

Supplementary material 1 (DOCX 1437 kb)

Supplementary material 2 (ZIP 194 kb)
