# Supplementary material for: Molecular dynamics re-refinement of two different small RNA loop structures using the original NMR data suggest a common structure
Source: J Biomol NMR. 2012 Jun 20;53(4):321–39. doi: 10.1007/s10858-012-9642-5 (PMC3405240; doi:10.1007/s10858-012-9642-5)
Supplement: Supplementary file 1 — Supplementary material 1 (DOCX 1437 kb) [file 10858_2012_9642_MOESM1_ESM.docx]

**Supplementary Information** to “Molecular dynamics re-refinement of two different small RNA loop structures using the original NMR data suggest a common structure”

*Journal of Biomolecular NMR* (2012) by NM Henriksen, DR Davis, and TE Cheatham, III.

Note that a separate files are available that contain the restraint data applied in the simulations and also AMBER formatted PDB files of the refined structures.

Specifically, these files are:

- ai5γ-DAR-DisAng.dat – distance and angle restraints in AMBER format.
- ai5γ-DAR-RDC.dat – RDC restraints in AMBER format.
- ai5γ-mDAR-DisAng.dat – distance and angle restraints in AMBER format for the modified (mDAR) refinement.
- ai5γ-mDAR-RDC.dat – distance and angle restraints in AMBER format for the modified (mDAR) refinement.
- PL-DAR-DisAng.dat – distance and angle restraints in AMBER format.
- PL-DAR-RDC.dat – RDC restraints in AMBER format.
- PL-mDAR-DisAng.dat – distance and angle restraints in AMBER format for the modified refinement.
- PL-mDAR-RDC.dat – RDC restraints in AMBER format for the modified refinement.
- 1R2P-DAR.pdb – PDBs for the ai5 γ re-refinement.
- 1R2P-DxAR.pdb – PDBs for the modified (mDAR) re-refinement of ai5γ
- 2F88-DAR.pdb – PDBs for the PL re-refinement.
- 2F88-DAR-heat.pdb – PDBs for the modified (mDAR) re-refinement of PL with heating

**NMR NMR x-ray re-refined NMR**

**ai5γ-D5, 1R2P PL-D5, 2F88 1KXK ai5γ-D5, mDAR PL-D5, mDAR**


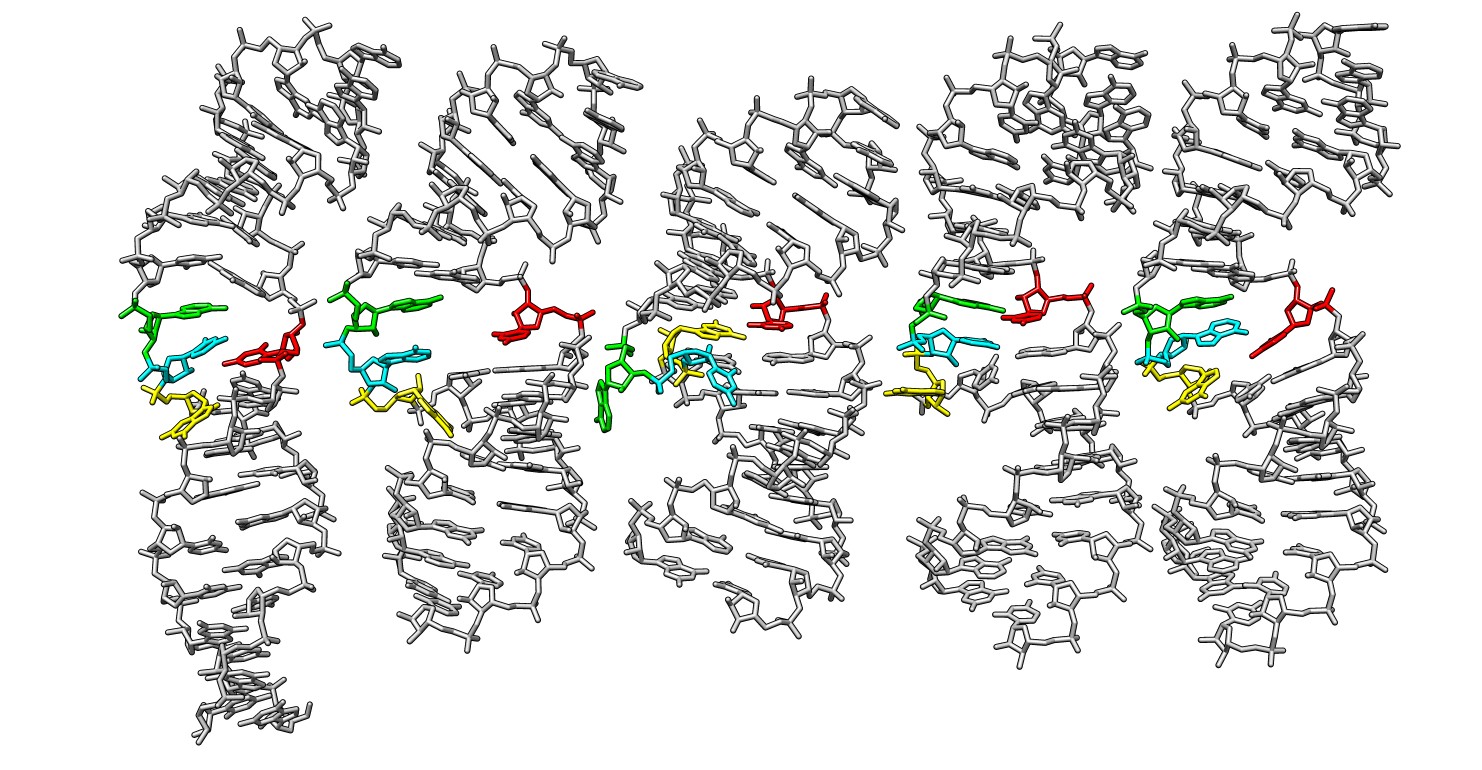


**Figure S1**: Molecular graphics representations of the heavy atoms of the previously and newly refined 34-residue portions of the domain 5 group II intron structures highlighting the differences in the bulge region. Shown are, from left to right, the earlier yeast ai5γ {Sigel, 2004 #3} (ai5γ-D5, PDB: 1R2P), the *Pylaiella littoralis* {Seetharaman, 2006 #2} (PL-D5, PDB: 2F88), a portion of the earlier crystal structure of ai5γ-D5 (PDB: 1KXK) {Zhang, 2002 #1}, the first structure of the mDAR/DxAR (*i.e.* refined including distance and angle restraints, residual dipolar coupling restraints, and removal of a few bad NOE restraints due to spin diffusion as per the main text) re-refinement of ai5γ, and the first structure of the mDAR/DAR-heat (*i.e.* refined with all of the restraint information and an additional heating step for better sampling as per the main text) re-refinement of PL-D5. G26 is colored in yellow, C25 in cyan, A24 in green and U9 is in red.

**Figure S2** (next page): RNAML 2-D schematics of the annotated secondary structure of the various older and re-refined structures highlighting the significant differences in the bulge regions.


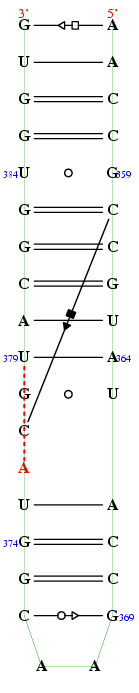

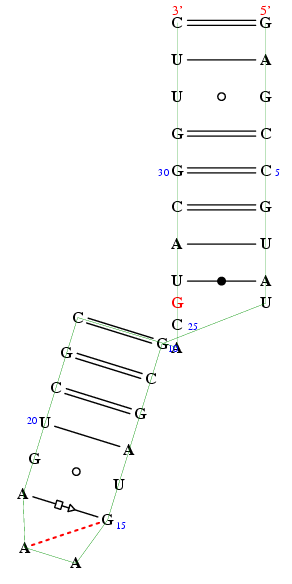
 **ai5γ-D5, PDB: 1R2P PL-D5, PDB: 2F88 PDB: 3IGI**


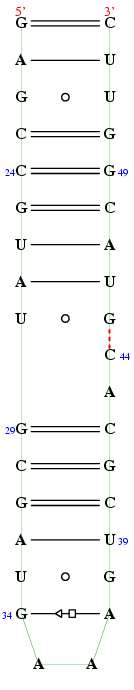


**PDB: 1KXK ai5γ-D5, re-refined PL-D5, re-refined**


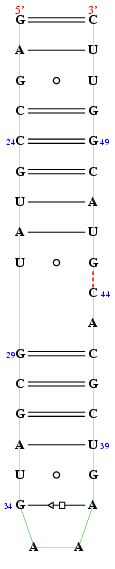

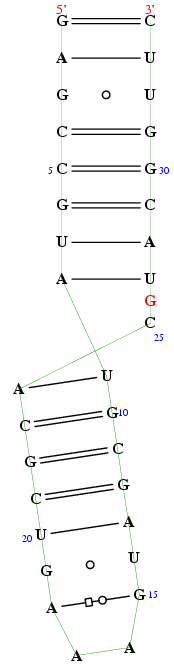

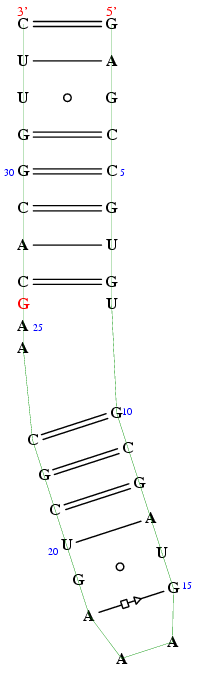


**
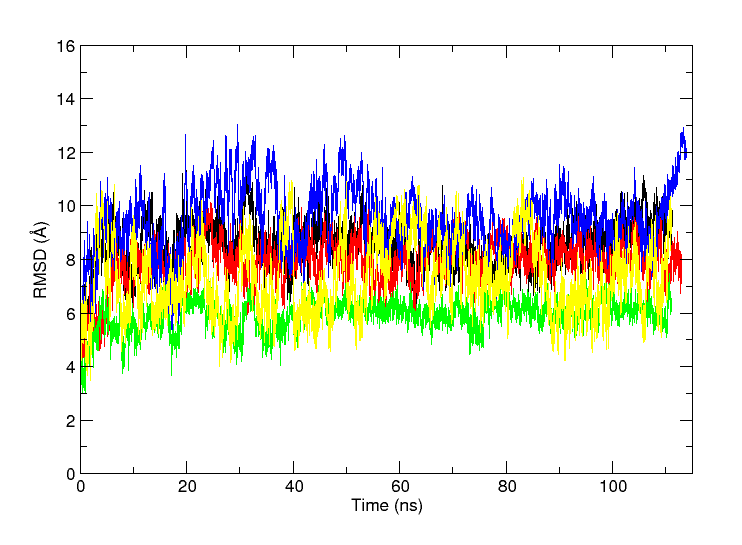
**


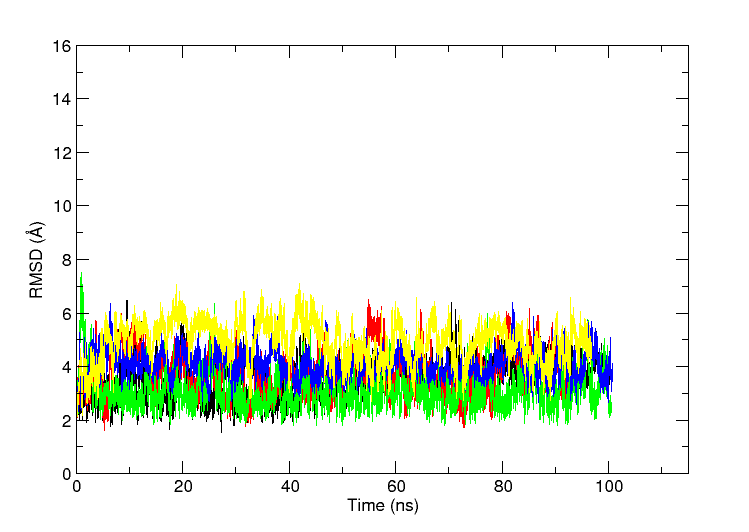


**Figure S3.** RMSD plot for the five unrestrained (UR) simulations of each PDB structure. PDB 1R2P or ai5γ_UR (top) and PDB 2F88 or PL_UR (bottom) simulations. RMSD values were calculated by fit to the initial structure. Although the RMSd values plateau, the RMSd values are relatively high indicating motion away from the starting structure.


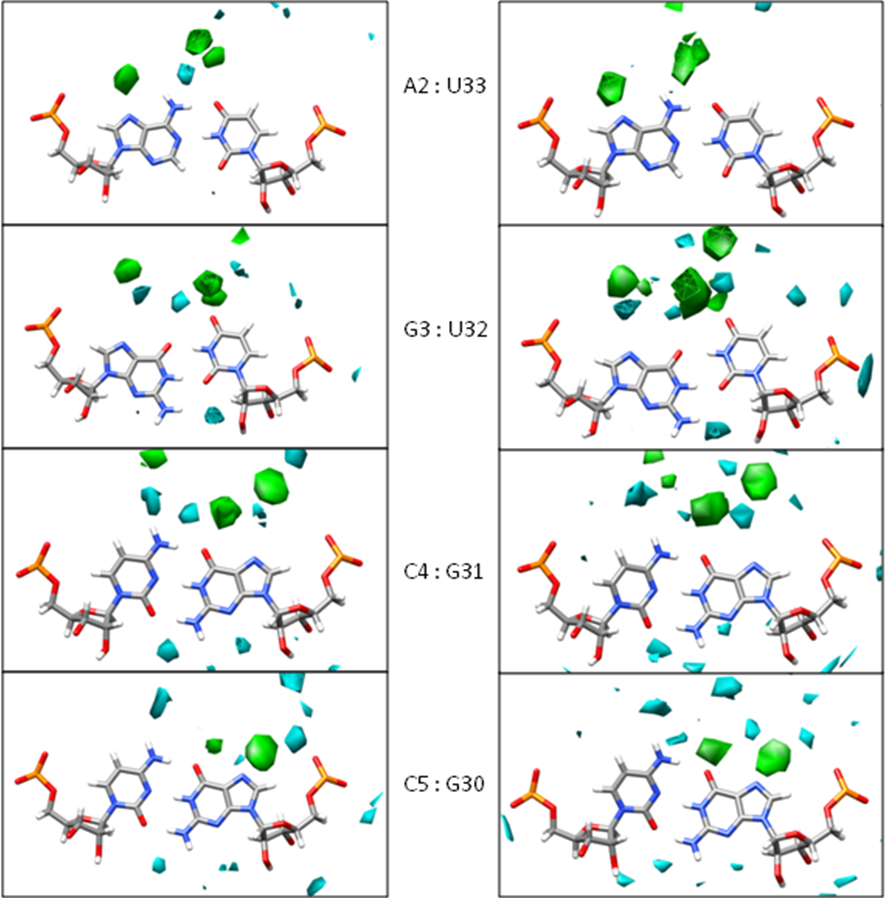


**Figure S4.** Comparison of water and Na^+^ densities in the major groove of selected base pairs for ai5γ_mDAR (left) and PL_mDAR (right).


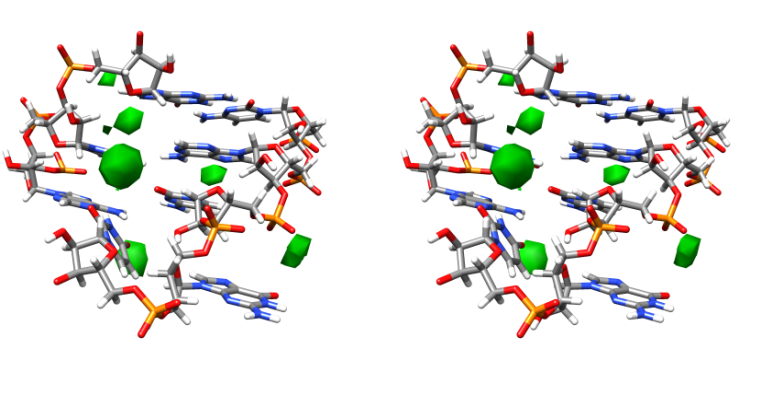


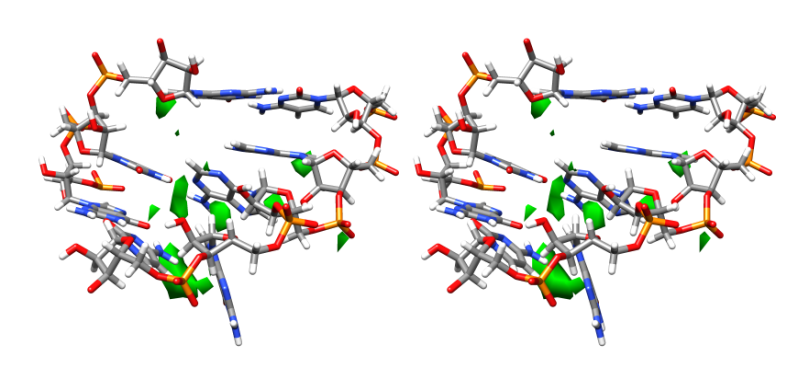

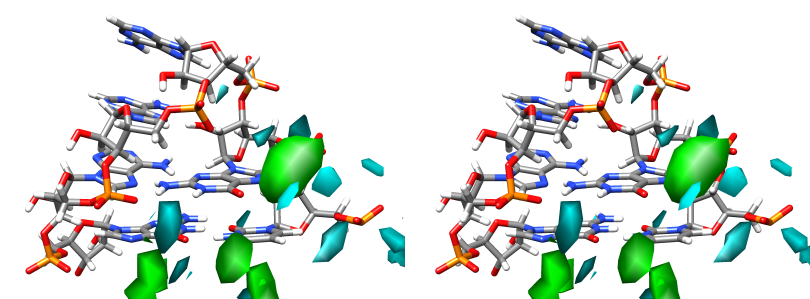

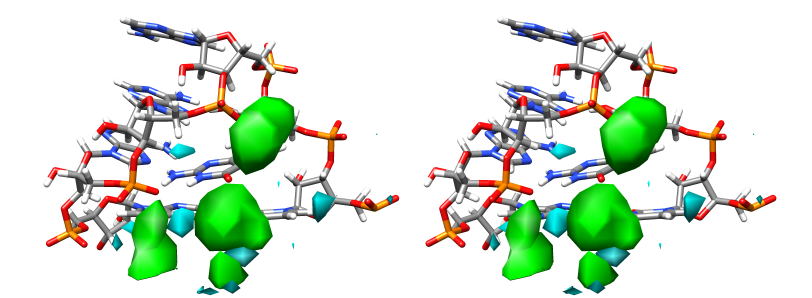


**Figure S5.** Stereo view of the Na^+^ density grid in the bulge region of ai5γ_mDAR (top) and PL_mDAR (bottom).

**Figure S6.** Stereo view of the Na^+^ density grid in the loop region of ai5γ_mDAR (top) and PL_mDAR (bottom).
